# The bidirectional effect of prelimbic 5-hydroxytryptamine type-4 (5-HT4) receptors on ACPA-mediated aversive memory impairment in adult male Sprague-Dawley rats

**DOI:** 10.22038/ijbms.2021.49501.11317

**Published:** 2021-06

**Authors:** Nargol Ahmadi-Mahmoodabadi, Masoumeh Emamghoreishi, Mohammad Nasehi, Mohammad-Reza Zarrindast

**Affiliations:** 1 Institute for Cognitive Science Studies, Tehran, Iran; 2 Department of Basic Sciences, Campus of Shahid Bahonar, Farhangian University of Shiraz, Shiraz, Iran; 3 Department of Pharmacology, School of Medicine and Department of Neuroscience, School of Advanced Medical Sciences and Technologies, Shiraz University of Medical Sciences, Shiraz, Iran; 4 Cognitive and Neuroscience Research Center, Tehran Medical Sciences, Islamic Azad University, Tehran, Iran; 5 Iranian National Center for Addiction Studies, Tehran University of Medical Sciences, Tehran, Iran; 6 Department of Pharmacology, School of Medicine, Tehran University of Medical Sciences, Tehran, Iran; 7 School of Cognitive Sciences, Institute for Research in Fundamental Sciences, Tehran, Iran

**Keywords:** ACPA, Pre-limbic cortex, Passive avoidance memory, RS23597-190, RS67333

## Abstract

**Objective(s):**

This study aimed at investigating the effect of serotonergic 5-HT4 receptor agonist/antagonist on memory consolidation deficit induced by ACPA (a potent, selective CB_1_ cannabinoid receptor agonist) in the pre-limbic (PL) cortex.

**Materials and Methods:**

We used the step-through passive avoidance test to evaluate memory consolidation of male Sprague-Dawley (SD) rats. Bilateral post-training microinjections of the drugs were done in a volume of 0.6 μl/rat into the PL area (0.3 μl per side).

**Results:**

The results showed a significant interaction between RS67333 hydrochloride (5-HT4 receptor agonist) or RS23597-190 hydrochloride (5-HT4 receptor antagonist) and ACPA on consolidation of aversive memory. RS67333 hydrochloride (0.5 μg/rat) enhanced consolidation of memory and its co-administration at the ineffective dose of 0.005 μg/rat with ineffective (0.001 μg/rat) or effective (0.1 μg/rat) doses of ACPA improved and prevented impairment of memory caused by ACPA, respectively. In other words, RS67333 had a bidirectional effect on ACPA-caused amnesia. While RS23597-190 hydrochloride had no effect on memory at the doses used (0.005, 0.01, 0.1, or 0.5 μg/rat); but its concomitant use with an effective dose of ACPA (0.1 μg/rat) potentiated amnesia. None of the drugs had an effect on locomotor activity.

**Conclusion:**

This study revealed that activation or deactivation of the 5-HT4 receptors in the PL may mediate the IA memory impairment induced by ACPA indicating a modulatory role for the 5-HT4 serotonergic receptors.

## Introduction

Serotonin or 5-hydroxytryptamine (5-HT), as an important brain neurotransmitter and neuromodulator, has a pivotal role in cognitive and non-cognitive functions (1). A broad range of studies presents the interplay between serotonergic neurotransmission and multiple other neurotransmitters including glutamate (Glu), γ-aminobutyric acid (GABA), dopamine (DA), acetylcholine (ACh), and cannabinoids (CBs). The interplay is involved in a variety of cognitive functions such as learning and memory processes (2, 3). The 5-hydroxytryptamine type-4 receptor (5-HT4 R) has a heterogeneous distribution pattern throughout the brain with high densities in limbic structures linked to memory and cognition (4, 5). Based on previous studies, 5-HT4 Rs could be a promising therapeutic target for the treatment of cognitive deficits (6, 7). The contribution of 5-HT4 Rs in learning and memory processes has been reviewed in the scientific literature (4, 8-10). 

The medial prefrontal cortex (mPFC) is one of the 5-HT4–enriched brain regions (11). The 5-HT_4_Rs have been expressed in about sixty percent of the PFC pyramidal neurons (12). There is now considerable evidence that mPFC plays an essential role in the consolidation of several types of memories, including modulation of formation and expression of fear memory (13). The pre-limbic (PL) area (a sub-region of the mPFC) (14) plays an important role in the modulation of emotional memory (15). PL integrates auditory and contextual input information and regulates the expression of fear memory via projections to the amygdala and hippocampus (16). Expression of the C-Fos & Arc gene has been reported in the PL region after inhibitory avoidance (IA) training, suggesting the vital role of mPFC in aversive learning mechanisms (17). The impairing impacts of cannabinoids on learning and memory have been the topic of extensive preclinical studies (18, 19). Cannabinoids influence cognitive function by interacting with neurochemical systems. Growing evidence indicates a potential interaction between serotonergic and endocannabinoid systems. In the PFC, alterations in serotonergic transmission occur in response to cannabinoid administrations (20, 21). Cannabinoid modulation of neuronal activity is largely mediated by cannabinoid type 1 receptors (CB_1_Rs) (22). CB_1_Rs are expressed in serotonergic fibers and synapses (3, 23). In addition, co-expression of CB_1_Rs and various types of serotonin receptors have been shown in the forebrain areas (24). Ferreira *et al*. showed that functional presynaptic CB_1_Rs were localized in frontocortical serotonergic nerve terminals and mediated as a modulator of serotonin neurotransmission (25). Furthermore, Balázsa *et al*., indicated that CB_1_Rs were implicated in nonsynaptic release modulation of ^[3H]^serotonin in the hippocampus (26). In the CNS, endocannabinoid (eCB) signaling is implicated in the release of serotonin (27, 28), as well as modulation of the activity and expression of different serotonin receptors (3, 29, 30). Likewise, serotonin receptor activation may evoke eCB release (31). Based on the above evidence, it is expected that serotonergic and eCB systems cross-control the activity of each other. However, there is no evidence supporting the presence of functional interaction between CB_1_Rs and 5-HT4 Rs in mediating the IA memory function in the PL. Therefore, this study was performed to evaluate the possible role of prelimbic 5-HT4 Rs in IA memory impairment caused by Arachidonylcyclopropylamide (ACPA; CB_1_R agonist) with the passive avoidance test (step-through type).

***Experimental procedures***

***Animals***

In order to perform the experiments, adult male rats with the scientific name of Rattus Norvegicus Allivias of Sprague-Dawley breed, weighing approximately 250 to 290 g at the time of surgery, were used. The animals were purchased from the Comparative and Experimental Medical Center, Shiraz University of Medical Sciences, Shiraz, Iran. Animals were kept (4/cage) in an animal house under the temperature of 22 ± 2 °C and 12/12 hr light/dark cycle (lights on at 07:00 hr) and had free access to water and food, except in the limited times of trials. Eight rats were used in each group and each animal was tested only once. The experiments were conducted during the light phase of the cycle. Animal care and behavioral tests were done in accordance with the Guide for the Care and Use of Laboratory Animals (32).

***Surgery***

All surgical procedures were performed under ketamine (70 mg/kg) – xylazine (7 mg/kg) anesthesia in a stereotaxic surgical apparatus (Stoelting Co, Illinois, USA) with a skull-flat orientation. Two stainless steel, 22-gauge guide-cannulas were bilaterally implanted 1 mm above the PL area according to the atlas of Paxinos and Watson. Stereotaxic coordinates of the PL were Anterior/Posterior (AP) equally +3.4 mm from the bregma, Medio/Lateral (ML) equally ±0.9 mm from the midline, and Dorso/Ventral (DV) equally −3 mm from the skull surface. The cannulas were secured to the skull bone using dental acrylic cement. Stainless steel stylets (dummy cannulae, 27 gauge) were inserted into the guide cannulae to prevent possible obstruction until the animals received the drugs. Following surgery, the animals were allowed to recover for at least 7 days before behavioral testing.

**Figure 1:**
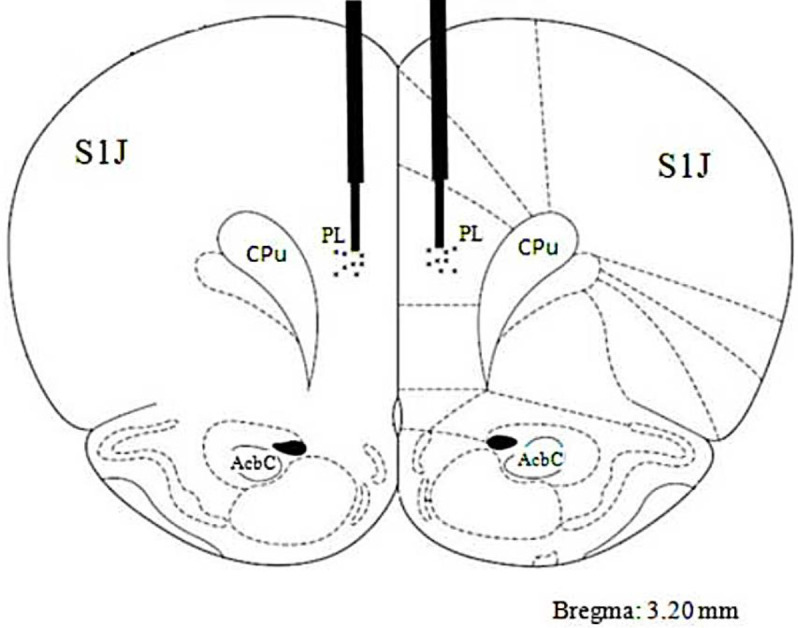
The approximate position of the tips of the infusion needles in the PL area for all intracerebral injections in the performed experiments on the coronal section which is taken from the Paxinos and Watson atlas

At the end of the tests, to ensure the accuracy of the infusion site (Figure 1), the animals were anesthetized with a high dose of ketamine/xylazine dilution. Next, methylene blue solution was injected into the PL area (1%, 0.3 μl/side) and animals were decapitated using a guillotine. The brains of animals were removed and stabilized in formalin solution (10%, 7 days) and sliced. The fixed brains were then sliced directly across the cannulae placements, and the placements were histologically verified using the rat brain atlas of Paxinos and Watson coordinates (33). Animals with incorrect cannulae placement (about 5% of total animals) were excluded from the analysis.

***Intra – PL injection***

In order to inject the drugs, rats were softly maintained by hand; then dummy cannulas were removed and substituted by 27 gauge infusion cannulas (1 mm below the tip of the guide cannulas). The infusion needle was joined to the Hamilton syringe (2-µl) via polyethylene tubing (PE-20). Intra-PL microinfusions of the drugs were performed with the volume of 0.6 μl per rat (0.3 μl per side) in a 60 sec period. Following the injections of drugs, the injectors were left in place for an additional 60 sec to allow the drugs to diffuse into the tissue. The interval time between the two injections was 5 min. One microinjection took about 9 min to complete (34, 35).

***Inhibitory (passive) avoidance task***

The passive avoidance (IA) task is an associative learning test, based on negative reinforcement used to assess memory (36). 

***Memory testing and apparatus***

**Figure 2:**
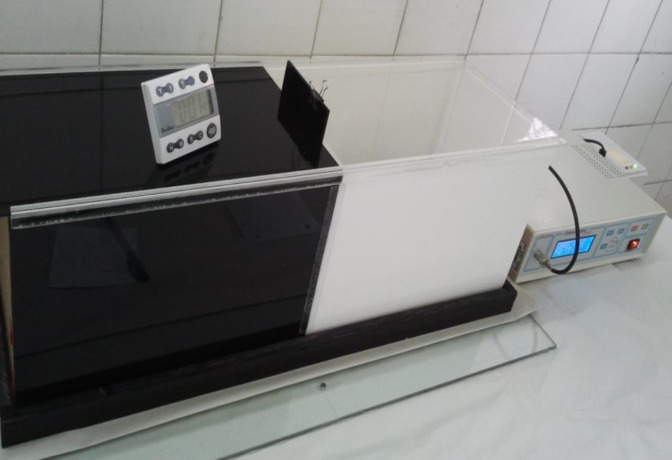
Passive-avoidance apparatus (step-through type)

The step-through passive avoidance device (Figure 2) included two same size chambers isolated by a sliding guillotine door (7 × 9 cm): a light chamber (30 cm × 20 cm × 20 cm) made of white opaque plexiglass and a dark chamber (30 cm × 20 cm × 20 cm) made of black opaque plexiglass, with parallel stainless steel grids on the floor which were connected to an isolated stimulator (Borj Sanat Co., Tehran, Iran). Alternative moderate unavoidable electrical shocks (Intensity =0.7 mA, Frequency=50 Hz, Duration=3 sec) were applied to the floor bars of the dark chamber, to create a foot shock. Memory evaluation was performed in habituation, training, and retention trial sessions that are based on the protocol applied in our prior research (35). In order to habituate to the experimental room, animals were placed in the experimental room for at least 30 min before the experiments. Next, each animal was softly left inside the light chamber and allowed for free exploration. Following 15 sec, the guillotine door separating the two chambers was completely opened and the latency of the animal to cross into the black chamber was recorded. After the rat entered with all four paws to the next chamber, the guillotine door was immediately closed and the animal was gently transferred into its cage. Rats that delayed more than 120 sec to enter the dark compartment were excluded from the experiments. 

After 30 min, during the training trial, the previous phase was repeated for each animal except that as soon as the animal entered the dark chamber it received an inevitable electrical foot shock through the grid floor of the chamber. Following 15 sec, the rat was taken out from the device and re-tested 2 min later in the same way. If the animal did not go into the black chamber in 120 sec, successful learning (IA response) would be recorded, otherwise, the animal received the shock again. Following obtaining a successful learning, the rat received micro-infusion of the drugs immediately after training, aimed to assess the impacts of the drugs on the consolidation of emotional memory.

Retention trial (24 hr after training) was conducted in the same way as the training session, exclude that the electrical shocks do not exert. The step-through latency (latency of entry within the black chamber) is defined as an index of the emotional memory consolidation. The cut-off time on the second day was 300 sec.

***Assessment of locomotor activity***

Motor activity was also assessed immediately after the retention trial session. In this regard, locomotion was recorded using an Animex activity meter device (Type DS, Farad electronics, Sweden). Rats separately were placed on the measurement platform and permitted to freely explore for a duration of 5 min. Each movement produced a signal which was automatically converted to numbers (37). Locomotor activity was evaluated by measuring the number of movements. Motor activity was evaluated by measuring the number of movements.

***Drugs***

The drugs which were utilized in this research were: ketamine hydrochloride/xylazine (Alfasan Chemical Co, Woerden, Holland) in order to anesthetize the animals. ACPA (arachidonylcyclopropylamide; a potent, selective agonist for CB_1_ receptor; in amounts of 0.001, 0.01, and 0.1 μg/rat), RS67333 hydrochloride (1-(4-amino-5-chloro-2-methoxyphenyl)-3-[1-butyl-4-piperidinyl]-1-propanone hydrochloride; a potent and highly selective partial agonist for 5-HT4 receptor; in amounts of 0.005, 0.01, 0.1, and 0.5 μg/rat), and RS23597-190 hydrochloride (3-(piperidine-1-yl) propyl 4-amino-5-chloro-2-methoxybenzoate hydrochloride; a high affinity, selective competitive antagonist for 5-HT4 receptor; in amounts of 0.005, 0.01, 0.1, and 0.5 μg/rat) acquired from Tocris (Tocris Bioscience Bristol, United Kingdom). All drugs were resolved in sterile 0.9% saline, with the exception of ACPA, which was prepared dissolved in anhydrous ethanol in the amount of 5 mg/ml and was diluted to the needed volume with saline. All of the drugs were made ready freshly just previous to testing. The injection timing and choice of drug dosages were based on the pilot and published studies in scientific journals (35, 38).

***Experimental design and drug treatment***

At first, the role of post-training, micro-infusion of the drugs in various dosages was separately examined on the consolidation of emotional memory in the pre-limbic area, and curves of dose-response were plotted. Next, the probable interplay between a sub-threshold dose of 5-HT4 receptors agonist or antagonist plus ACPA in various dosages was evaluated. Eight rats were employed in each experimental group and each rat was examined just once. Bilateral intra-PL microinjection of the drugs was conducted immediately after a training session in a volume of 0.6 μl/rat (0.3 μl/side). The animals received one or two injections in the experiments. The Interval time between two drug injections was 5 min. Behavioral tests (passive avoidance & locomotor activity) were assessed in all experiments, as described in previous sections. The test session was performed 24 hr later, following the drug microinjection(s). 

***Experiment 1: Evaluating the effect of post-training intra-PL microinjections of RS67333 hydrochloride (5-HT4 receptor agonist) and RS23597-190 hydrochloride (5- HT***_4_ ***receptor antagonist) on IA memory consolidation***

Ten groups of animals (n=8/group) received saline (0.6 μl/rat, two groups), RS67333 (a 5-HT4 Rs agonist; 0.005, 0.01, 0.1, or 0.5 μg/rat) or RS23597-190 (a 5-HT4 Rs antagonist; 0.005, 0.01, 0.1, or 0.5 μg/rat) immediately after training.

***Experiment 2: Evaluating the effect of post-training intra-PL microinjection of saline, RS67333 hydrochloride, or RS23597-190 hydrochloride on IA memory impairment induced by ACPA***

Twelve groups of animals were utilized. The animals were distributed into three four-group sets. Rats were initially injected with saline (0.6 μl/rat), the subthreshold dose of RS67333 (0.005 μg/rat), or RS23597-190 (0.5 μg/rat) immediately after training. Then after 5 min, rats were injected with vehicle (0.6 μl/rat) or different doses of ACPA (0.001, 0.01, and 0.1 μg/rat).

***Statistical analysis***

Kolmogorov–Smirnov test showed normal distributions of data in all groups. Therefore, data were analyzed using one- or two-way analysis of variance (ANOVA). One-way ANOVA was performed to assess the individual effects of the drugs. Two-way ANOVA was accomplished for the statistical assessment of possible interactions between the drugs. Subsequently a significant F value, Tukey’s *post-hoc* analysis was done to evaluate paired-group comparisons. The results were presented as mean ± S.E.M. and *P*<0.05 was considered as a statistically significant difference. SPSS software ver. 19 was used for statistical analyses.

## Results

***Post-training intra-PL microinjection effects of RS67333 and RS23597-190 hydrochloride on memory consolidation and exploratory behaviors***

**Figure 3:**
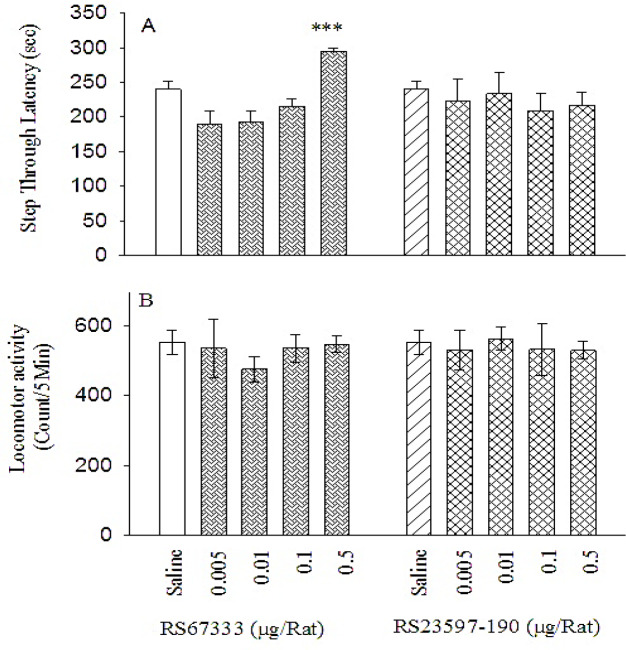
Effects of post-training intra-PL microinjections of RS67333 and RS23597-190 on IA memory consolidation (A) and locomotor activity (B) in rats. Ten groups of animals (n=8/group) received either saline (0.6 μl/rat, two groups), or different doses of RS67333 (5- HT_4_ receptor agonist; 0.005, 0.01, 0.1, and 0.5 μg/rat) and RS23597-190 (5- HT_4_ receptor antagonist; 0.005, 0.01, 0.1, and 0.5 μg/rat), immediately after training. Step through latency and locomotor activity were evaluated in all groups after 24 hr. Each column shows mean ± SEM. ***P<0.001, as compared with the saline control group

One-way ANOVA analysis revealed that local intra-PL administrations of RS67333 altered consolidation of IA memory [F(4,35) = 11.042, *P* =0.000 <0.05, (Figure 3A, left panel)], while it did not alter locomotor activity behavior [F(4,35) = 0.408, *P*=0.801 > 0.05, (Figure 3B, left panel)]. Moreover, Tukey’ *post-hoc* test showed that RS67333 at dose of 0.5 μg/rat significantly increased the step-through latency in passive avoidance learning task, during the test session. Based on the results, it appears that RS67333 has an enhancing effect on aversive memory consolidation. Moreover, one-way ANOVA indicated that post-training intra-PL administration of RS23597-190 at different doses (0.005, 0.01, 0.1, and 0.5 μg/rat) could neither alter the IA memory consolidation [F(4,35) = 0.248, *P*=0.909 > 0.05, (Figure 3A, right panel)], nor the locomotor activity behavior [F(4,35) = 0.100, *P*=0.982 > 0.05, (Figure 3B, right panel)], suggesting that RS23597-190 alone at the applied doses did not affect memory consolidation.

***Effect of post-training intra-PL microinjection of RS67333 hydrochloride or RS23597-190 hydrochloride on the ACPA induced IA memory consolidation deficit***

**Figure 4:**
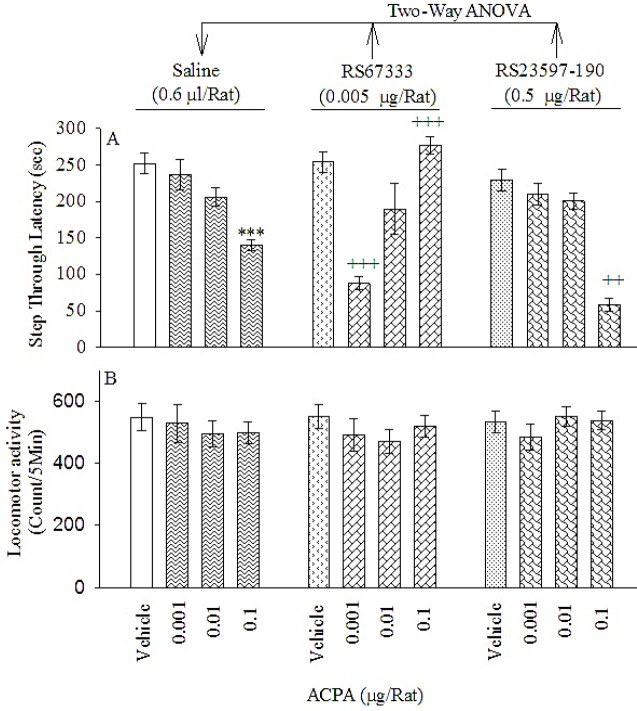
Effects of post-training intra-PL microinjections of ACPA on IA memory consolidation (panel A) and locomotor activity (panel B) in the presence and absence of RS67333 (0.005 μg/rat) or RS23597-190 (0.5 μg/rat). Three four-group sets of rats were utilized. Rats were injected with saline (0.6 μl/rat), the subthreshold dose of RS67333 (0.005 μg/rat) or RS23597-190 (0.5 μg/rat) plus vehicle or ACPA at different doses (0.001, 0.01, or 0.1 μg/rat). Step through latency (STL) and locomotor activity were evaluated in all groups, after 24 hr. Each column shows mean±SEM. ***P<0.001, different from vehicle/saline group. ++*P*<0.01 and +++*P*<0.001 different from respective ACPA/saline groups

One-way ANOVA demonstrated that ACPA significantly altered memory consolidation [F(5,42) = 13.770,, *P*=0.000 < 0.05, (Figure 4A, left panel)] but did not affect locomotor activity [F(5,42) = 1.199, *P*=0.326 > 0.05, (Figure 4B, left panel)]. Tukey’s *post-hoc* analysis showed that ACPA at a dose of 0.1 μg/rat impaired IA memory consolidation. 

Furthermore, two-way ANOVA revealed a significant interaction between RS67333 plus ACPA on memory consolidation [F dose (3,56) = 8.882, *P*=0.000 < 0.05; F drug (1,56) = 0.256, *P*=0.609 > 0.05; F dose × drug (3,56) = 21.588, *P*=0.000 < 0.05, (Figure 4A, middle panel)], but not locomotor activity [F dose (3,56) = .793, *P*=0.503 > 0.05; F drug (1,56) = 0.102, *P*=0.751 > 0.05; F dose × drug (3,56) = 0.168, *P*=0.917 > 0.05, (Figure 4B, middle panel)]. Tukey’s *post-hoc* test showed that the ineffective doses of ACPA (0.001 μg/rat) and RS67333 (0.005 μg/rat) when combined, significantly potentiated the emotional memory impairment. Curiously, RS67333 (0.005 μg/rat) when combined with the effective dose of ACPA (0.1 μg/rat), prevented the effect of the latter, on the test day as compared with the respective control groups. Our results showed that co-administration of RS67333 and ACPA produced a bidirectional effect upon IA memory consolidation, in the passive avoidance (PA) task.

In addition, two-way ANOVA showed a significant interaction between RS23597-190 plus ACPA on memory consolidation [F dose (3,56) = 43.195, *P*<0.0005; F drug (1,56) = 12.442, *P*<0.001; F dose × drug (3,56) = 2.980, *P*<0.039, (Figure 3A, right panel)], but not locomotor activity [F dose (3,56) = .221, *P*=0.882; F drug (1,56) = 0.081, *P*=0.777; F dose × drug (3,56) = 0.661, *P*=0.580, (Figure 3B, right panel)]. Tukey’s *post-hoc* test showed that post-training co-administration of a subthreshold dose of RS23597-190 (0.5 μg/rat) plus the higher dose of ACPA (0.1 μg/rat) strengthened the ACPA effect.

## Discussion

This study revealed that 5-HT4 Rs agonist (RS67333) in the presence of CB_1_ receptor agonist (ACPA) produced bidirectional effects on the consolidation of aversive memory in the PL area. The enhancing effect of RS67333 on memory consolidation is in agreement with the studies that showed that 5-HT4 Rs agonists improved the learning and memory process (4, 10, 39-41). However, some studies have reported that RS67333 impaired consolidation of memory (38, 42, 43). 

Based on several lines of studies, serotonin influenced neuronal plasticity and memory formation through multiple intracellular signaling pathways which are implicated in diverse effectors such as cyclic adenosine monophosphate (cAMP) (8, 44). Emerging evidence suggests a flexible mechanism for 5-HT4 Rs to modulate synaptic transmission and neuronal excitability in the PFC networks (7). The 5-HT4 Rs are G_s_-protein-coupled receptors and positively coupled to adenylyl cyclase. It seems that agonist activation of these receptors by engaging the downstream signaling cascades probably activates the cAMP formation (45, 46) and participates in the new memory formation (9). In addition, 5-HT4 Rs agonists improved facilitation of the various neurotransmitter releases in the brain structures linked to memory function and enhanced synaptic transmission which might have affected the development of memory (47, 48). 5-HT4 Rs also represented constitutive (ligand-independent) activity which elucidates the differences between expected and observed effects of agonists and antagonists of the 5-HT4 Rs (48). 5-HT4 Rs splice variants have been identified both in rodents and humans (49, 50) with structural differences. It might be influenced and contributed to their functional diversity and involved in the fine-tuning of the receptor coupling to G-protein subtypes. Some of splice variants of 5-HT4 Rs are able to activate both G_αi/o_- and G_αs_-proteins (51). These variants may interact with distinct or overlapping signaling machinery leading to differential intracellular responses (9). It may be possible that RS67333’s effect in this paradigm is being mediated in part or significantly by its action on other receptors such as sigma receptors which are also known to ameliorate anxiety-related responses and affect PFC neural transmission and memory function (52, 53). It has been shown that RS67333 (54) and RS23597-190 (55) had a high affinity for sigma-1 binding sites. It may indicate that the highest tested dose of RS23597-190 was showing an effect similar to the lower dose of RS67333, despite their antithetical pharmacological action. Therefore, RS67333 like other 5-HT4 agonists may interact with other receptors and its effect might not be mediated solely by action on 5-HT4 Rs (56).

The present results also revealed that there is a significant interaction between RS67333 or RS23597-190 plus ACPA on memory consolidation. Furthermore, RS67333 potentiated or reversed ACPA response (a bidirectional effect). In accordance with our findings, it has been shown that 5-HT4 Rs agonists such as RS67333 reversed memory impairment induced by diverse classes of pharmacological agents in different behavioral tasks (41, 57-61). However, some reports are describing the intensifying effect of RS67333 on ACPA-induced amnesia (38, 62). 

The mechanisms underlying the roles of cannabinoid-based drugs are not fully known. Increasing evidence indicates the bidirectional interaction between the cannabinoid and the serotonergic systems which employed different direct and indirect mechanisms and brain structures (3, 23, 25). This is partially rationalized because of: 1. A high level of functional overlapping between these two systems in the regulation of several physiological functions (3), 2. Extensively overlapping distribution pattern of CB_1_ and 5-HTRs in the brain (63), and 3. Engaging both of these systems in creating the connections and the maturation of brain neocortical circuitry as well as in neuromodulation of glutamatergic and GABAergic transmission in the PFC (64). Co-expression of 5-HT and CB_1_Rs has been shown in the brain, representing possible interactions between them (24). Cannabinoids display CBR-independent activity and target non- CB_1_/CB_2_ GPCRs that may contribute to the pharmacological actions of CBs (65). On the other hand, some studies indicated the ability of CB_1_Rs to form homo- and heteromeric complexes with the 5-HTRs (66, 67). These interactions mediate different aspects of CB_1_R function. Based on previous studies, CB_1_Rs have unusual properties such as the dual capacity for inhibition or activation of adenylate cyclase by linking to G_i/o _(68) or G_s_ proteins (69) and influencing the intracellular signaling pathways. It is the potential of CB_1_Rs to modulate the activity of the other receptor systems. In addition, CB_1_Rs are mainly expressed in the presynaptic glutamatergic and GABAergic neurons (70). It has been reported that CB_1_R activation is involved in the modulation of synaptic plasticity by controlling PKA activity in the GABAergic cells (71). On the other hand, dual effects of 5-HT4 Rs agonists have been shown on the GABAergic inhibitory postsynaptic currents (IPSCs) in the PFC pyramidal neurons. Its activation-induced enhancement or reduction of the GABAergic evoked currents (72) depending on the protein kinase A activation (7) and participated in modulation of synaptic transmission and neuronal excitability. In addition, PKA is known as a cAMP-dependent protein kinase and works through the cAMP signaling pathway (73). Therefore, it is possible that co-activation of CB_1_ and 5-HT4 Rs has influenced the intracellular cAMP accumulation and participated in the memory process through engaging downstream signaling pathways. It can be said that this response “bidirectional effects of 5-HT4 Rs agonist in the presence of CB_1_Rs agonist on memory consolidation” is likely the result of CB_1_R switching from Gi to Gs signaling pathways and vice versa (69, 74).

## Conclusion

In summary, this study showed that: 1. Intra-PL injection of RS67333 but not RS23597-190 increased IA memory consolidation., 2. There is a significant interaction between RS67333 or RS23597-190 plus ACPA on memory consolidation., 3. RS67333 potentiated or reversed ACPA response (a bidirectional effect)., 4. RS23597-19 intensified ACPA-induced impairment of memory consolidation. We suggest that activation or deactivation of 5-HT4 Rs in the PL area, presumably was involved in memory impairment induced by ACPA in the step-through IA task. Future studies are required to uncover the details.

## References

[1] Green AR (2006). Neuropharmacology of 5-hydroxytryptamine. Br J Pharmacol.

[2] Dhonnchadha BÁN, Cunningham KA (2008). Serotonergic mechanisms in addiction-related memories. Behav Brain Res.

[3] Haj-Dahmane S, Shen RY (2011). Modulation of the serotonin system by endocannabinoid signaling. Neuropharmacology.

[4] King MV, Marsden CM, Fone KCF (2008). A role for the 5-HT1A, 5-HT4 and 5-HT6 receptors in learning and memory. Trends Pharmacol Sci.

[5] Bockaert J, Claeysen S, Compan V, Dumuis A (2004). 5-HT4 Receptors. Curr Drug Targets CNS Neurol Disord.

[6] Murphy SE, Wright LC, Browning M, Cowen PJ, Harmer CJ (2019). A role for 5-HT4 receptors in human learning and memory. Psychol Med.

[7] Cai X, Flores-Hernandez J, Feng J, Yan Z (2002). Activity-dependent bidirectional regulation of GABAA receptor channels by the 5-HT4 receptor-mediated signalling in rat prefrontal cortical pyramidal neurons. J Physiol.

[8] Perez-Garcia G, Meneses A (2008). Memory formation, amnesia, improved memory and reversed amnesia: 5-HT role. Behav Brain Res.

[9] Ahmad I, Nirogi R (2011). 5-HT4 Receptor agonists for the treatment of alzheimer’s disease. Neurosci Med.

[10] Orsetti M, Dellarole A, Ferri S, Ghi P (2003). Acquisition, retention, and recall of memory after injection of RS67333, a 5-HT4 receptor agonist, into the nucleus basalis magnocellularis of the rat. Learn Mem.

[11] Lucas G, Compan V, Charnay Y, Neve RL, Nestler EJ, Bockaert J (2005). Frontocortical 5-HT4 receptors exert positive feedback on serotonergic activity: Viral transfections, subacute and chronic treatments with 5-ht4 agonists. Biol Psychiatry.

[12] Feng J, Cai X, Zhao JH, Yan Z (2001). Serotonin receptors modulate GABAA receptor channels through activation of anchored protein kinase C in prefrontal cortical neurons. J Neurosci.

[13] Euston DR, Gruber AJ, McNaughton BL (2012). The role of medial prefrontal cortex in memory and decision making. Neuron.

[14] Vertes RP (2006). Interactions among the medial prefrontal cortex, hippocampus and midline thalamus in emotional and cognitive processing the rat. Neurosci.

[15] Stern CAJ, Gazarini L, Vanvossen AC, Hames MS, Bertoglio LJ (2014). Activity in prelimbic cortex subserves fear memory reconsolidation over time. Learn Mem.

[16] Sotres-Bayon F, Sierra-Mercado D, Pardilla-Delgado E, Quirk GJ (2012). Gating of fear in prelimbic cortex by hippocampal and amygdala inputs. Neuron.

[17] Zhang Y, Fukushima H, Kida S (2011). Induction and requirement of gene expression in the anterior cingulate cortex and medial prefrontal cortex for the consolidation of inhibitory avoidance memory. Mol Brain.

[18] Lichtman AH, Varvel SA, Martin BR (2002). Endocannabinoids in cognition and dependence. Prostaglandins Leukot Essent Fatty Acids.

[19] Tan H, Lauzon NM, Bishop SF, Chi N, Bechard M, Laviolette SR (2011). Cannabinoid transmission in the basolateral amygdala modulates fear memory formation via functional inputs to the prelimbic cortex. J Neurosci.

[20] Egerton A, Allison C, Brett RR, Pratt JA (2006). Cannabinoids and prefrontal cortical function: insights from preclinical studies. Neurosci Biobehav Rev.

[21] Maejima T, Masseck OA, Mark MD, Herlitze S (2013). Modulation of firing and synaptic transmission of serotonergic neurons by intrinsic G protein-coupled receptors and ion channels. Front Integr Neurosci.

[22] Howlett AC, Breivogel CS, Childers SR, Deadwyler SA, Hampson RE, Porrino LJ (2004). Cannabinoid physiology and pharmacology: 30 years of progress. Neuropharmacology.

[23] Haring M, Marsicano G, Lutz B, Monory K (2007). Identification of the cannabinoid receptor type 1 in serotonergic cells of raphe nuclei in mice. Neuroscice.

[24] Hermann H, Marsicano G, Lutz B (2002). Coexpression of the cannabinoid receptor type 1 with dopamine and serotonin receptors in distinct neuronal subpopulations of the adult mouse forebrain. Neurosci.

[25] Ferreira SG, Teixeira FM, Garcao P, Agostinho P, Ledent C, Cortes L (2012). Presynaptic CB (1) cannabinoid receptors control frontocorticals serotonin and glutamate release – species differences. Neurochem Int.

[26] Balazsa T, Biro J, Gullai N, Ledent C, Sperlagh B (2008). CB1-cannabinoid receptors are involved in the modulation of non-synaptic [3H]serotonin release from the rat hippocampus. Neurochem Int.

[27] Aso E, Renoir T, Mengod G, Ledent C, Hamon M, Maldonado R (2009). Lack of CB1 receptor activity impairs serotoninergic negative feedback. J Neurochem.

[28] Tzavara ET, Davis RJ, Perry KW, Li X, Salhoff C, Bymaster FP (2003). The CB1 receptor antagonist SR 141716A selectively increases monoaminergic neurotransmission in the medial prefrontal cortex: implications for therapeutic actions. Pharmacol Biochem Behav.

[29] Zavitsanou K, Wang H, Dalton VS, Nguyen V (2010). Cannabinoid administration increases 5HT1A receptor binding and mRNA expression in the hippocampus of adult but not adolescent rats. Neuroscince.

[30] Moranta D, Esteban S, Garcia-Sevilla JA (2009). Chronic treatment and withdrawal of the cannabinoid agonist WIN 55,212-2 modulate the sensitivity of presynaptic receptors involved in the regulation of monoamine syntheses in rat brain. Naunyn-Schmiedeberg’s Arch Pharmacol.

[31] Best AR, Regehr WG (2008). Serotonin evokes endocannabinoid release and retrogradely suppresses excitatory synapses. Neuroscince.

[32] N.R.C (2011). Guide for the Care and Use of Laboratory Animals.

[33] Paxinos G, Watson C (2007). The rat brain in stereotaxic coordinates.

[34] Rasekhi K, Oryan S, Nasehi M, Zarrindast MR (2014). Involvement of the nucleus accumbens shell glutamatergic system in ACPA-induced impairment of inhibitory avoidance memory consolidation. Behav Brain Res.

[35] Ahmadi-Mahmoodabadi N, Nasehi M, Emam Ghoreishi M, Zarrindast M-R (2016). Synergistic effect between prelimbic 5-HT3 and CB1 receptors on memory consolidation deficit in adult male Sprague–Dawley rats: An isobologram analysis. Neuroscience.

[36] Ogren SO, Stiedl O (2015). Passive Avoidance. Encyclopedia of Psychopharmacol.

[37] Ueki A, Miwa Ch, Miyoshi K (1994). Impairment in the acquisition of passive and active avoidance learning tasks due to bilateral entorhinal cortex lesions. Neurol Sci.

[38] Chegini HR, Nasehi M, Zarrindast MR (2014). Differential role of the basolateral amygdala 5-HT3 and 5-HT4 serotonin receptors upon ACPA-induced anxiolytic-like behaviors and emotional memory deficit in mice. Behav Brain Res.

[39] Lamirault L, Simon H (2001). Enhancement of place and object recognition memory in young adult and old rats by RS 67333, a partial agonist of 5-HT4 receptors. Neuro Pharmacol.

[40] Meneses A (2007). Stimulation of 5-HT1A, 5-HT1B, 5-HT2A/2C, 5-HT3 and 5-HT4 receptors or 5-HT uptake inhibition: short- and long-term memory. Behav Brain Res.

[41] Freret T, Bouet V, Quiedeville A, Nee G, Dallemagne P, Rochais C (2012). Synergistic effect of acetylcholinesterase inhibition (donepezil) and 5-HT(4) receptor activation (RS67333) on object recognition in mice. Behav Brain Res.

[42] Nasehi M, Davoudi K, Ebrahimi-Ghiri M, Zarrindast M-R (2016). Interplay between serotonin and cannabinoid function in the amygdala in fear conditioning. Brain Res.

[43] Nasehi M (2014). The modulatory effect of CA1 5HT 4 receptors on memory acquisition deficit induced by harmaline. J Paramed Sci.

[44] Polter AM, Li X (2010). 5-HT1A receptor-regulated signal transduction pathways in brain. Cell Signal.

[45] Bockaert J, Claeysen S, Compan V, Dumuis A (2008). 5-HT4 receptors: History, molecular pharmacology and brain functions. Neuropharmacology.

[46] Steven CL, Yan L, Alan LP, Elena D, Gennady S, Connie S (2015). Serotonergic regulation of prefrontal cortical circuitries involved in cognitive processing: A review of individual 5 HT receptor mechanisms and concerted effects of 5 HT receptors exemplified by he multimodal antidepressant vortioxetine. ACS Chem Neurosci.

[47] Seyedabadi M, Fakhfouri G, Ramezani V, Mehr SE, Rahimian R (2014). The role of serotonin in memory: interactions with neurotransmitters and downstream signaling. Exp Brain Res.

[48] Pytliak M, Vargova V, Mechirova V, Felsoci M (2011). Serotonin receptors from molecular biology to clinical applications. Physiol Res.

[49] Coupar IM, Desmond PV, Irving HR (2007). Human 5-HT4 and 5-HT7 Receptor splice variants: Are they important. Curr Neuropharmacol.

[50] Claeysen S, Sebben M, Becamel C, Bockaert J, Dumuis A (1999). Novel brain-specific 5-HT4 receptor splice variants show marked constitutive activity: role of the C-terminal intracellular domain. Mol Pharmacol.

[51] Pindon A, Hecke GV, Gompel PV, Lesage AS, Leysen JE, Jurzak M (2002). Differences in signal transduc-tion of two 5-HT4 receptor splice variants: compound specificity and dual coupling with gαs and gαi/o-proteins. Molecular Pharmacology.

[52] Ji LL, Peng JB, Fu CH, Cao D, Li D, Tong L (2016). Activation of Sigma-1 receptor ameliorates anxiety-like behavior and cognitive impairments in a rat model of post-traumatic stress disorder. Behav Brain Res.

[53] Zhang Y, Lu W, Wang Z, Di Z, Wang G, Aa J (2020). Reduced neuronal cAMP in the nucleus accumbens damages blood-brain barrier integrity and promotes stress vulnerability. Biol Psychiatry.

[54] Eglen RM, Bonhaus DW, Johnson LG, Leung E, Clark RD (1995). Pharmacological characterization of two novel and potent 5-HT4 receptor agonists, RS 67333 and RS 67506, in vitro and in vivo. Br J Pharmacol.

[55] Bonhaus DW, Loury DN, Jakeman LB, Hsu SA, To ZP, Leung E (1994). [3H]RS-23597-190, a potent 5-hydroxytryptamine4 antagonist labels sigma-1 but not sigma-2 binding sites in guinea pig brain. J Pharmacol Exp Ther.

[56] Wawra M, Fidzinski P, Heinemann U, Mody I, Behr J (2014). 5-HT4-Receptors modulate induction of long-term depression but not potentiation at hippocampal output synapses in acute rat brain slices. PLoS One.

[57] Eriksson T, Delagrange P, Spedding M, Popoli M, Mathé A, Ögren S (2012). Emotional memory impairments in a genetic rat model of depression: involvement of 5-HT/MEK/Arc signaling in restoration. Mol Psychiatry.

[58] Matsumoto M (2001). Evidence for involvement of central 5-HT4 receptors in cholinergic function associated with cognitive processes: behavioral, electrophysiological, and neurochemical studies. J Pharmacol Exp Ther.

[59] Shen F, Smith JAM, Chang R, Bourdet DL, Tsuruda PR, Obedencio GP (2011). 5-HT4 Receptor Agonist Mediated Enhancement of Cognitive Function in vivo and Amyloid Precursor Protein Processing in vitro: A Pharmacodynamic and Pharmacokinetic Assessment. Neuropharmacology.

[60] Lelong V, Lhonneur L, Dauphin F, Boulouard M (2003). BIMU 1 and RS 67333, two 5-HT4 receptor agonists, modulate spontaneous alternation deficits induced by scopolamine in the mouse. Naunyn Schmiedebergs Arch Pharmacol.

[61] Micale V, Leggio GM, Mazzola C, Drago F (2006). Cognitive Effects of SL6 0155 a Serotonin 5-HT4 Receptor Partial Agonist, in Animal Models of Amnesia. Brain Res.

[62] Nasehi M, Kafi F, Khakpai F, Zarrindast MR (2015). Involvement of the serotonergic system of the ventral hippocampus (CA3) on amnesia induced by ACPA in mice. Behav Brain Res.

[63] Zarate J, Churruca I, Echevarria E, Casis L, De Jesus ML, Del Burgo LS (2008). Immunohistochemical localization of CB 1 cannabinoid receptors in frontal cortex and related limbic areas in obese Zucker rats: Effects of chronic fluoxetine treatment. Brain Res.

[64] Daubert EA, Condron BG (2010). Serotonin: A regulator of neuronal morphology and circuitry. Neurosci.

[65] Oz M (2006). Receptor-independent actions of cannabinoids on cell membranes: Focus on endocannabinoids. Pharmacol Ther.

[66] Hudson BD, Hébert TE, Kelly ME (2010). Ligand- and heterodimer-directed signaling of the CB(1) cannabinoid receptor. Mol Pharmacol.

[67] Busquets-Garcia A, Bains j, Marsicano G (2018). CB1 Receptor signaling in the brain: Extracting specificity from ubiquity. Neuropsychopharmacol Rev.

[68] Howlett AC, Barth F, Bonner TI, Cabral G, Casellas P, Devane WA (2002). Classification of cannabinoid receptors. Pharmacol Rev.

[69] Kumar KK, Shalev-Benami M, Robertson MJ, Hongli Hu, Banister SD, Hollingsworth SA (2019). Structure of a signaling cannabinoid receptor 1-g protein complex. Cell.

[70] Bosier B, Muccioli GG, Hermans E, Lambert DM (2010). Functionally selective cannabinoid receptor signalling: Therapeutic implications and opportunities. Biochem Pharmacol.

[71] Heifets BD, Chevaleyre V, Castillo PE (2008). Interneuron activity controls endocannabinoid mediated presynaptic plasticity through calcineurin. PNAS.

[72] Celada P, Puig MV, Artigas F (2013). Serotonin modulation of cortical neurons and networks. Front Integr Neurosci.

[73] Søberg K, Moen LV, Skålhegg BS, Laerdahl JK (2017). Evolution of the cAMP-dependent protein kinase (PKA) catalytic subunit isoforms. PLoS One.

[74] Turu G, Szlo L, Hunyady L (2010). Signal transduction of the CB1 cannabinoid receptor. J Mol Endocrinol.

